# Inadequate Choline Intake in Pregnant Women in Germany

**DOI:** 10.3390/nu14224862

**Published:** 2022-11-17

**Authors:** Merle Roeren, Anna Kordowski, Christian Sina, Martin Smollich

**Affiliations:** Institute of Nutritional Medicine, University Hospital Schleswig-Holstein, Campus Luebeck, University of Luebeck, 23538 Luebeck, Germany

**Keywords:** choline, pregnancy, vegan, vegetarian, omnivorous, adequate intake

## Abstract

Choline is an essential nutrient that is involved in various developmental processes during pregnancy. While the general adequate choline intake (AI) for adults has been set at 400 mg/day by the European Food Safety Authority (EFSA), an AI of 480 mg/day has been derived for pregnant women. To date, the choline intake of pregnant women in Germany has not been investigated yet. Therefore, in this survey, the total choline intake from dietary and supplementary sources in pregnant women was estimated using an online questionnaire. A total of 516 pregnant women participated in the survey, of which 283 met the inclusion criteria (13 to 41 weeks of gestational age, 19–45 years). 224 (79%) of the participants followed an omnivorous diet, 59 (21%) were vegetarian or vegan. Median choline intake was 260.4 (±141.4) mg/day, and only 19 women (7%) achieved the adequate choline intake. The median choline intake of omnivores was significantly higher than that of vegetarians/vegans (269.5 ± 141.5 mg/day vs. 205.2 ± 101.2 mg/day; *p* < 0.0001). 5% (13/283) of pregnant women took choline-containing dietary supplements. In these women, dietary supplements provided 19% of the total choline intake. Due to the importance of choline for the developmental processes during pregnancy, the study results prove the urgent need for an improved choline supply for pregnant women.

## 1. Introduction

Choline is an essential nutrient being involved in many physiological processes in the human body. It is a constituent of the neurotransmitter acetylcholine and it modulates, as a precursor to the cell membrane components phosphatidylcholine and sphingomyelin, membrane integrity, transmembrane signaling, myelination, cell growth, and cell division [1]. Moreover, choline acts as a methyl group donor via the synthesis of s-adenosylmethionine [2], thereby essentially contributing to epigenetic methylation reactions, DNA stability, and cellular metabolism [3,4,5].

Correspondingly, higher phosphatidylcholine intake is associated with lower risk of incident dementia and better cognitive performance [6], and dietary choline intake has been suggested to play a relevant role in the prevention of cognitive decline [7,8], poststroke cognitive impairment [9], and poststroke depression [10]. Even more, choline has recently been found to be neuroprotective against prenatal alcohol exposure-related brain structure deficits in humans [11,12]. Respective effects of choline may, at least in part, be mediated by a functional interaction with vitamin B12 [13]. Dietary choline can be converted to trimethylamine (TMA) by the colonic microbiota, with TMA being further metabolized to trimethylamine-*N*-oxide (TMAO) in the liver [14]. The role of choline-derived TMAO for cardiovascular health is subject to controversial discussions [15].

A choline deficiency has not been reported at a population level, but has been observed in experimental settings and total parenteral nutrition only [16,17,18]. However, inadequate choline intake has been linked to non-alcoholic fatty liver disease (NAFLD), skeletal muscle atrophy, neurodegenerative diseases, and several ocular diseases including retinal hemorrhage, glaucoma, and dry eye syndrome [19,20]. Furthermore, a variety of choline-related inherited metabolic diseases has been described [21].

Even though choline, in principle, can be synthesized de novo through the methylation of phosphatidylethanolamine, the endogenous synthesis is not sufficient to meet the physiological choline requirements [22]. Therefore, choline must at least in part be acquired from dietary sources. Choline is mainly found in foods of animal origin such as eggs, poultry, and meat [23,24]. Plant-based foods such as legumes, leafy greens, and nuts, contain only small amounts of choline [25]. For an Australian cohort, it has been shown that eggs, red meat, nuts, legumes, and dairy account for 50% of the choline intake, with eggs alone contributing 17% [26]. In a randomized cross-over trial, the consumption of two eggs significantly increased the plasma choline level of adult [27]. Accordingly, it is difficult to meet the adequate choline intake especially for vegetarians and vegans [28,29].

During pregnancy and lactation, the dietary requirements for choline are substantially higher than for non-pregnant women, since the fetus and the infant accumulate choline at the expense of maternal stores [30,31]. Therefore, the adequate intake (AI) for choline in pregnant women has been set at 480 mg/day by the European Food Safety Authority (EFSA), compared to 400 mg/day for non-pregnant adults [32]. A growing body of evidence strongly suggests that choline plays a crucial role during neuronal fetal development [33], e.g., by contributing to fetal brain and memory development [34], acetylcholine biosynthesis, and neuronal cell signaling [25,35,36,37]. Accordingly, an increased choline intake during pregnancy probably improves the neurocognitive outcomes in the offspring [4,35,38]. Most recently, a meta-analysis found that a low maternal choline intake is not only associated with impaired child neurocognition and neurodevelopment, but also with an increased risk of neural tube defects [39]. However, substantial evidence from randomized-controlled trials investigating the prenatal effects of choline is still lacking, especially regarding both possible dose response-relationships between maternal choline intake and child neurocognitive outcomes and potential interactive effects of the two methyl-donor nutrients choline and folate [40].

Taking together the major relevance of a sufficient choline intake for the fetal neuronal development, the elevated choline requirements during pregnancy, and the poor choline content of plant-based foods, it can be supposed that pregnant women following a vegetarian or vegan diet have a high risk of not achieving the recommended AI for choline. Moreover, choline is absent in most dietary supplements marketed for pregnant women, with a median daily choline dose of only 25 mg [41,42].

Most surveys during pregnancy suggest that choline intakes are considerably below the AI [23], but the clinical assessment of choline status remains difficult [43]. In Germany, the choline intake of pregnant women has never been assessed systematically before. Therefore, we estimated the dietary and supplementary choline intake of pregnant women in Germany with an online survey. To detect possible subgroup differences, both omnivores and vegetarians/vegans have been included.

## 2. Materials and Methods

### 2.1. Study Design and Participants

For this online survey, pregnant women were recruited via social media in November and December 2021, using a questionnaire on the SurveyMonkey platform. The inclusion criteria were a gestational age of 13 weeks or higher, and an age between 19 and 45 years. In total, 516 subjects started the questionnaire, of whom 283 met the inclusion criteria (Figure S1). The sample size calculation was based on epidemiological data: In Germany, the population size is approx. 500,000 pregnant women in the second and third trimester of the pregnancy. With a confidence level set at 90% and a margin of error at 5%, the sample size has been calculated *n* = 273.

The participants were informed about the purpose of the study and their formal consent was collected before they started the questionnaire. Ethical review and approval were not required for the study in accordance with the local legislation and institutional requirements.

### 2.2. Questionnaire

The study was conducted in Germany and the questionnaire was in German language. It comprised 30 questions that were further divided into four different parts: health and pregnancy; dietary supplement use; a food frequency questionnaire (FFQ) which specified 60 choline-containing food items or groups; and questions about the sociodemographic background of the participants (Figure S2). 

The FFQ was based on the National Health and Nutrition Examination Survey (NHANES) questionnaires [44] and the Project Viva FFQ [45], which have been used for the estimation of choline intake before [46]. The questionnaire used in the present study was designed to assess the choline intake from the diet within the previous week. Pictures of hand portion sizes were added to visualize the portion size and to have a standard for the subsequent evaluation. The FFQ focused on choline-containing foods only. To assess the dietary choline intake, we referred to Zeisel’s measurements [47]. Using a drop-down list, respondents were able to determine the respective number of foods/food groups consumed during the previous week. Subsequently, the study population was categorized based on their dietary pattern (omnivore vs. vegetarian/vegan).

Finally, participants documented their intake of dietary supplements during the pregnancy (trademark, duration, dosing).

### 2.3. Data Analysis

The individual dietary choline intake was calculated by multiplying the frequency of consumption per week by consumed amounts of all the assessed food products. The concentration of choline in every respective food item was taken from previous studies [47]. To assess the daily intake, total weekly choline intake of each individual was divided by seven. The median and the interquartile range (IQR) of the entire cohort were calculated. To estimate the daily choline intake from dietary supplements, the participants answered the questions about the frequency and the dosage of the supplements they took.

For all data, statistical analyses were performed using GraphPad Prism. To test for the normal distribution, the Shapiro–Wilk test was applied. Since the normal distribution could not be assumed, non-parametric tests were used to statistically analyze the choline intake. Median values of total choline intake and dietary choline intake were calculated and presented in milligrams per day, with min to max error bars. Choline intake was compared to the adequate choline intake for pregnant women (480 mg/day).

Multivariate analysis for confounding variables was performed by linear regression. 

The statistical comparison for the analysis of the different groups (omnivore to vegetarian/vegan to all) was carried out using a Kruskal–Wallis test with Dunn’s test for multiple analyses; when only 2 groups were compared, the Mann–Whitney was used. We adjusted for outliers in the whole population referring to the daily intake. Outliers above the 95% percentile and below the 5% percentile were excluded (in total 10 data points, 8 omnivore and 2 vegetarian/vegan).

## 3. Results

### 3.1. Study Population

The baseline characteristics of the study population (*n* = 283) are shown in Table 1. Most participants were aged between 26–35 years (234/283), lived with their partner or family (278/283), and had a university degree (144/283). 56 (20%) participants changed their diet due to the pregnancy, and 16 (6%) took choline-containing dietary supplements. Among participants taking choline-containing dietary supplements, only three received a recommendation for it. Referring to health in pregnancy, the mean number of days of feeling nauseous was 50, with 54 (19%) of participants reporting weight loss due to vomiting.

In the context of qualitative representativeness, our sample is representative for pregnant women in Germany regarding age distribution and living situation. Regarding education, women with a university degree (51%) are overrepresented in our sample.

59 (21%) women followed a vegetarian/vegan diet, while 224 (79%) were omnivorous. These two groups differed across health and sociodemographic characteristics. Vegetarian/vegan women were more likely to be older, more educated, and more likely to take choline-containing dietary supplements (8% vs. 5%). Moreover, they were less likely to lose weight due to vomiting (14% vs. 21%). In contrast, omnivorous women were less likely to change their diet for the pregnancy (20% vs. 76%). No-one in the vegetarian/vegan group received a recommendation to take choline-containing dietary supplements.

### 3.2. Total Choline Intake

For total choline intake, the estimated choline intake from both diet and dietary supplements were added. Only 7% (19/283) of participants achieved the adequate choline intake of 480 mg/day. The median choline intake was 263.5 ± 147.8 mg/day. 93% of omnivores (208/224) and 95% of vegetarians/vegans (56/59) had an inadequate choline intake when applying the choline AI (median: 274.3 ± 156 mg/day and 209.2 ± 107.7 mg/day, respectively).

**Figure 1:**
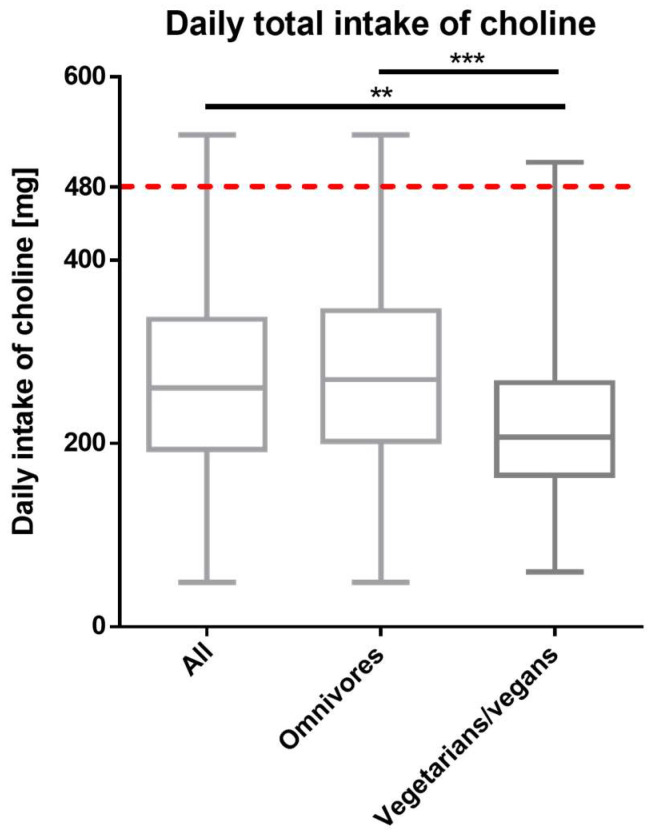
Total (dietary and supplementary) daily choline intake in all (*n* = 273), omnivorous (*n* = 217) and vegetarian/vegan (*n* = 56) participants (outliers excluded). The red dotted line represents the choline AI of 480 mg/day. Data are presented as median with whiskers from ‘min to max’ and was analyzed with Kruskal–Wallis test following the Dunn’s test for multiple comparisons (** adjusted *p* value < 0.01; *** adjusted *p* value < 0.001).

After excluding outliers (choline intake > 558.70 mg/day), the median choline intake remained below the AI with 260.4 ± 141.4 mg/day for all, 269.5 ± 141.5 for omnivores, and 205.2 ± 101.2 mg/day for vegetarians/vegans (Figure 1). The difference in daily choline intake between omnivores and vegetarians/vegans was statistically significant (*p* < 0.001) (Figure 1). Calculating the odds ratio (OR), the vegetarian/vegan group had 30% lower odds of meeting the AI than the omnivorous group (95% CI 0.21–2.35, Table 2).

As a result of the multivariate analysis for possible confounders (age, education, nauseous days, diet), apart from the diet, the age of 36–40 years was the only confounding factor.

### 3.3. Dietary Choline Intake

**Figure 2:**
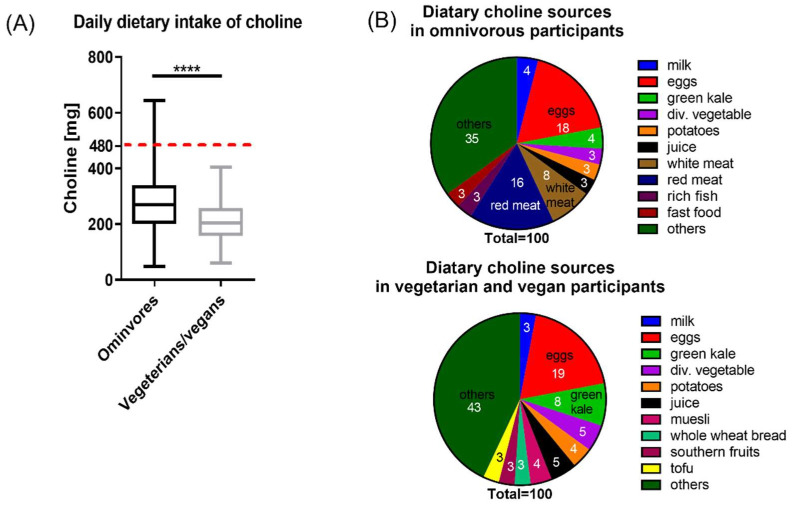
Dietary choline intake. (**A**) Daily dietary choline intake in omnivorous and vegetarian/vegan participants. The AI of 480 mg/day is represented by the red dotted line. Data are presented as median with whiskers from ‘min to max’ and was analyzed with Mann–Whitney test (**** *p* < 0.0001). (**B**) Dietary choline sources in omnivorous and vegetarian/vegan participants. Data are shown as parts of 100, numbers in the graph indicate the mean contribution of the given food to the total dietary choline intake.

Applying the AI for pregnant women, only 7% of the participants achieved an adequate choline intake. The median dietary choline intake was 267.8 ± 137.7 mg/day for omnivorous women and 204.4 ± 99.5 mg/day for vegetarian/vegan women (*p* < 0.0001) (Figure 2A).

The minimal dietary choline intake was 49 mg per day, being higher among vegetarians/vegans than among omnivores (60.06 mg/day vs. 48.69 mg/day). Diet contributed most to total choline intake in both groups, with differences between omnivores and vegetarians/vegans. The main sources of choline in omnivores were eggs (56.7 mg/day), red meat (48.2 mg/day), and white meat (25.1 mg/day); whereas, in the vegetarians/vegans, eggs, green kale, and fruit juice contributed the most with 42.7 mg/day, 19.1 mg/day, and 11.8 mg/day, respectively (Figure 2B). 3–4% of the total choline intake via food was provided by milk and potatoes in both groups.

### 3.4. Choline Intake from Dietary Supplements 

**Figure 3:**
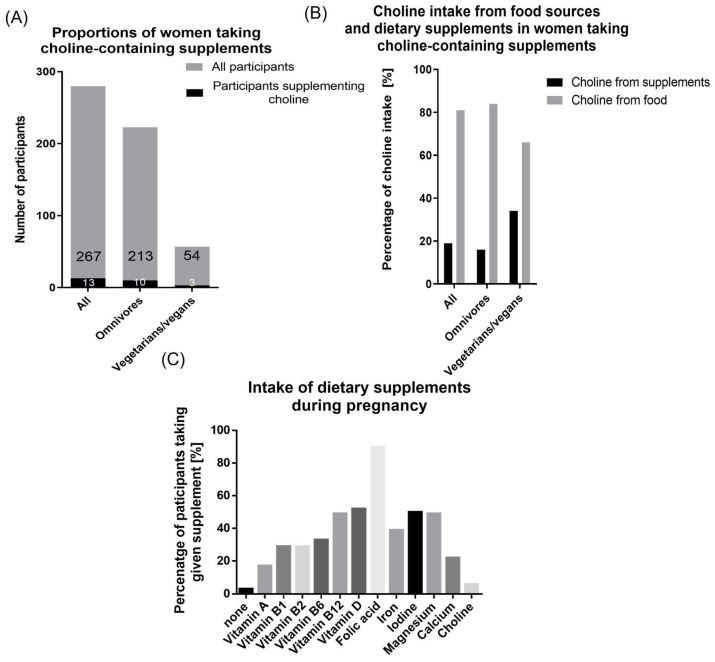
Intake of dietary supplements. (**A**) Proportion of participants taking choline-containing dietary supplements in all (13/267), omnivorous (10/213), and vegetarian/vegan (3/54) participants (**B**) Percentage of choline coming from food sources and dietary supplements in participants supplementing choline (all *n* = 13; omnivore *n* = 10, vegetarian/vegan *n* = 3) (**C**) Intake of dietary supplements during pregnancy. Percentage of participants taking given supplement alone or as a part of a prenatal vitamin complex supplement.

In total, 13/283 (5%) participants reported to take choline-containing dietary supplements (omnivores: *n* = 10; vegetarians/vegans: *n* = 3, Figure 3A). In these women, dietary supplements accounted for 19% of total choline intake. Differentiating between dietary habits, choline-containing supplements contributed 16% to the total choline intake of omnivores (mean supplementary choline intake: 100.94 mg/day), but 34% (mean supplementary choline intake: 126.67 mg/day) to the total choline intake of vegetarians/vegans (Figure 3B). 

### 3.5. Dietary Supplement Use

274/283 (97%) participants took any dietary supplement during pregnancy. 90% of the women took folic acid alone or as part of a prenatal vitamin complex (Figure 3C). Vitamin D was supplemented by 52%, iodine by 50%, and magnesium and vitamin B12 by 49% each.

## 4. Discussion

### 4.1. Main Finding

Our study was the first to estimate the dietary and supplemental choline intake of pregnant women in Germany, demonstrating that 93% of pregnant women do not meet the adequate choline intake, with vegetarian/vegan women having an even lower chance of achieving the adequate intake. Moreover, taking dietary supplements does not substantially improve the situation.

### 4.2. Previous Findings on Choline Intake

As early as 1998, choline was recognized as an essential nutrient, and the adequate choline intake has been set for both the general population and pregnant women by the Institute of Medicine (IOM) [48]. In contrast, until now, no respective recommendations have been published by the German Society of Nutrition.

Data on the dietary and supplementary choline intake of healthy adults are only available for North America and few European countries; respective data for Germany are lacking. Most surveys during pregnancy suggest that the AI of choline is met by few women only [23]. Accordingly, the choline intake of healthy, non-pregnant women has recently been estimated at 291 mg/day (France), 285 mg/day (Greece), 334 mg/day (The Netherlands), 294 mg/day (UK), and 362 mg/day (Australia) [26,49]. For Germany, total choline intake estimates have only been published for children and adolescents with an average intake in females ranging from 151–295 mg/day [50]. With the estimated median choline intake in our survey being 260.4 mg/day, our results are consistent with previous findings as they are both close to the estimated choline intake from neighboring countries with similar dietary habits (291–374 mg/day) [49], and very similar to the estimated choline intake of German female adolescents (295 mg/day) [50]. The result of the multivariate analysis indicating that the age of 36–40 years is the only confounding factor (apart from the diet) can be interpreted as a statistical artefact, as it cannot be plausibly explained by physiological/psychological hypotheses and previous studies.

Investigating the choline intake during pregnancy, systematic data for European countries are lacking almost completely. The only data published so far come from a Latvian survey showing an average choline intake among pregnant women of 336 mg/day [51], which too is in line with our results. Moreover, our finding that 93% of pregnant women in Germany do not reach the adequate choline intake is in line with similar data from the US with 91% of the pregnant women not meeting the AI for choline [52].

### 4.3. Studies on Choline-Containing Dietary Supplements

A randomized controlled trial assessed the effect of third trimester maternal choline supplementation (930 mg/day vs. 480 mg/day) on child memory at 7 years of age. Both groups were above the adequate intake levels in Germany [32]. Children of higher supplemented mothers scored better results than children in the control group with lower choline doses. Another study investigating the effect of prenatal choline supplementation (500 mg/day vs. 25 mg/day) on maternal and fetal biomarkers of choline metabolism measured higher plasma concentrations of free choline, betaine, dimethylglycine, phosphatidylcholine, and sphingomyelin among higher supplemented women [53]. Moreover, pregnancy-related metabolic adaptions were supported in this trial. These findings indicate that even the choline AI set for pregnant women by the EFSA may not be sufficient for optimal offspring neurodevelopment. However, in both studies the sample size was relatively small which makes it difficult to draw generalized conclusions. Furthermore, compared to the supplements used in our study, the dietary supplements used in these trials by far exceeded the choline doses used in Germany.

Most women in our study supplemented folic acid (FA) during their pregnancy. Recent studies suggest that imbalances between FA and other methyl-donor nutrients involved in one-carbon metabolism can determine the pregnancy outcomes [54] and metabolic adaptions [55]. Folic acid and choline play critical roles in the production of S-adenosylmethionine (SAM), a key modulator of DNA methylation [56]. However, in contrast to FA, choline is absent in most prenatal dietary supplements in Germany. This aspect is also observed in our study, since most of the women took folic acid-containing dietary supplements while most of these supplements did not contain any choline.

### 4.4. Implications

Our results for the first time demonstrate that the choline intake of pregnant women in Germany generally does not meet the recommendations for an adequate intake, with vegetarian and vegan women having an even lower chance of achieving the AI for choline. Moreover, taking dietary supplements does not improve the situation. Thus, it can be concluded that currently neither the majority of pregnant women, nor health care professionals, nor manufacturers of dietary supplements are aware of choline being a critical nutrient in pregnancy. Furthermore, our results underline the imbalance of folic acid and choline intake in pregnant women in Germany. Several implications arise from our results.

First, obviously, it is very difficult to meet the recommended AI for choline with a regular, omnivorous diet, and it is even more so for pregnant women following a vegetarian or vegan diet. Therefore, it might be suggested to advice pregnant women to change their dietary habits, thereby improving their choline intake. This approach, however, is hardly practicable considering the low success rates of general dietary recommendations. Even more, pregnant women sticking to a vegetarian or vegan diet due to ethical reasons are very unlikely to switch to an omnivorous, egg and meat-containing diet just to increase their choline supply. On the other hand, small dietary changes even of omnivorous women will not be able to substantially increase their choline intake.

Second, the awareness that choline might be an essential nutrient in pregnancy should be raised, both among pregnant women and healthcare professionals, including gynecologists, midwives, general practitioners, and pharmacists. The urgent need for improved choline provision for pregnant women, both through individual counselling and public health interventions, has been emphasized by other authors before [57]. This goal, however, is unlikely to be achieved through general information campaigns, but rather through targeted training and continuing medical education programs. If choline-specific dietary recommendations prove to be insufficient or unsuccessful, the intake of choline-containing dietary supplements might be considered. Particularly, respective supplements may be useful in vegetarian/vegan women and in women suffering from nausea and vomiting during pregnancy. Since vegetarian and vegan diets are increasingly common in pregnant women, there is an increased risk that the maternal choline supply will deteriorate as a result.

Third, the results of our survey suggest that it might be useful to add sufficient amounts of choline to products that are advertised for pregnant women. As shown here, most dietary supplements used do not contain any choline. If they do contain choline, the respective concentrations are too low to substantially improve the choline supply of the pregnant women.

Finally, fourth, more randomized controlled trials are needed to further specify the health benefits for the offspring resulting from an improved maternal choline intake. The adequate choline intake recommendations are not derived from randomized controlled trials but estimated from epidemiological data only. Therefore, it must be kept in mind that not meeting the recommended AI not necessarily means that the respective individual (or her offspring) is insufficient of choline or even suffering from clinically relevant deficiency.

### 4.5. Strengths and Limitations

It is a particular strength of our study that it makes an important contribution to putting the focus on the choline supply of pregnant women. The results presented here are the first to estimate the total choline intake of pregnant women in Germany, both from dietary and supplementary sources and differentiating between omnivorous and vegetarian/vegan diets. A major strength of our methodology is the semiquantitative questionnaire with hand portion pictures that enabled improved estimation of dietary intakes.

Regarding the limitations, it has to be considered that our data are based on a non-probability convenience sample rather than a representative population-based sample. With this type of sampling, the generalizability of our results is limited to populations that share similar characteristics with our sample. Therefore, it remains questionable whether the results would be similar in a representative sample. Due to the case number calculation, the quantitative representativeness of our results is given. In terms of qualitative representativeness, our sample is representative for pregnant women in Germany regarding age distribution and living situation. However, regarding education, women with a university degree (51%) are overrepresented in our sample, as due to census data only 28% of women < 60 years hold a university degree. Thus, the choline intake of pregnant women without a university degree might differ from the results presented here. 

In this context, a selection bias might be relevant, too. Recruitment was done through social media, so pregnant women without appropriate media use were not reached. Women with a heightened interest in nutritional issues and dietary supplements probably preferentially participated in the survey. When interpreting the data, it must be noted that the number of subjects in some subgroups was too small to obtain statistically meaningful results. This problem must be addressed with appropriately powered follow-up studies.

Additionally, the choline content of several foods was unknown since it has never been analyzed and published. As a result, some dietary choline sources were not named so that the final result might be lower. Specifically, the FFQ used in our study did not include vegan and vegetarian meat or dairy alternatives which might have affected the estimated choline intake especially in vegetarians and vegans.

Since the FFQ method is widely used in nutrition surveys, its inherent limitations are well-known and have been discussed in detail elsewhere [58]. Additionally, some participants obviously misread the instructions and filled in the questionnaire not for a week but rather for a whole month. In order to attenuate this error, we excluded outliers as indicated. An alternative approach for food intake assessment might have been repeated dietary recalls or records; however, an FFQ is more achievable in a large cohort and within the given time frame [59], even more, it is less prone to over- or underestimating the food intake than other methods [60].

Of course, the results may not be transferred to other countries without further ado, since not only dietary habits may differ, but also the medical counselling of pregnant women, the market situation of dietary supplements, the health policies and public opinion regarding food fortification, and the women’s attitude towards taking dietary supplements.

Finally, any evaluation of choline intake must be done with caution, as intake below the AI not necessarily indicates a health-affecting deficiency [49].

## 5. Conclusions

Due to the relevance of choline for fetal development, and considering our results that suggest an inadequate choline intake in pregnant women in Germany, efforts to encourage the increased intake of choline-rich foods and/or choline-containing dietary supplements during pregnancy might be useful. This is especially true for pregnant women who follow a vegetarian or vegan diet. Moreover, further research is necessary to define optimal choline requirements in pregnancy.

## Figures and Tables

**Table 1:** Descriptive statistics of the study population. Selected characteristics of the study cohort (*n* = 283) stratified by dietary patterns. Values are absolute number and percentages unless stated otherwise within diet group according to the categories in the first column.

	Vegetarian/Vegan *n* = 59 (21%)	Omnivore *n* = 224 (79%)	Total *n* = 283 (100%)
Age (years)			
19–25	6 (10%)	11 (5%)	17 (6%)
26–30	21 (36%)	101 (45%)	122 (43%)
31–35	25 (42%)	87 (39%)	112 (40%)
36–40	6 (10%)	23 (10%)	29 (10%)
41–45	1 (2%)	2 (1%)	3 (1%)
≥46	0	0	0
Living situation			
Living alone	1 (2%)	2 (1%)	3 (1%)
Living with partner	33 (56%)	107 (48%)	140 (49%)
Living with family	24 (41%)	114 (51%)	138 (49%)
Others	1 (2%)	1 (1%)	2 (1%)
Level of education			
Secondary school	1 (2%)	3 (1%)	4 (1%)
Secondary modern school	9 (15%)	47 (21%)	56 (20%)
Grammar school	10 (17%)	64 (29%)	74 (26%)
University	38 (64%)	106 (47%)	144 (51%)
Others	1 (2%)	4 (2%)	5 (2%)
Median (IQR) gestational week	31 (17)	24 (14.75)	25 (15)
Parity			
1	29 (49%)	105 (47%)	134 (47%)
2	17 (29%)	70 (31%)	87 (31%)
≥3	13 (22%)	48 (22%)	62 (22%)
Median (IQR) days feeling nauseous	60 (78)	32 (75)	35 (74)
Weight loss due to vomiting	8 (14%)	46 (21%)	54 (19%)
Changed diet for pregnancy	11 (76%)	45 (20%)	56 (20%)
Choline-containing supplement intake	5 (8%)	11 (5%)	16 (6%)
Choline recommendation by doctor, alternative practitioner, or nutritionist	0 (0%)	3 (1%)	3 (1%)

**Table 2:** Participants who met the adequate choline intake (AI) of 480 mg/d.

	All	Omnivores	Vegetarians/Vegans
AI	19 (7%)	16 (7%)	3 (5%)
Total	283	224	59
Odds ratio		1.0	0.70 (0.21–2.35)

## Data Availability

Data available upon request from the corresponding author.

## Supplementary Materials

The following supporting information can be downloaded at: https://www.mdpi.com/article/10.3390/nu14224862/s1, Figure S1: Flow Chart. Figure S2: Original questionnaire in German language with an example of the drop-down list used for the food frequency questionnaire.

## References

[1] Zeisel S.H., Niculescu M.D. (2006). Perinatal Choline Influences Brain Structure and Function. Nutr. Rev..

[2] Zeisel S. (2017). Choline, Other Methyl-Donors and Epigenetics. Nutrients.

[3] Jiang X., Yan J., West A.A., Perry C.A., Malysheva O.V., Devapatla S., Pressman E., Vermeylen F., Caudill M.A. (2012). Maternal choline intake alters the epigenetic state of fetal cortisol-regulating genes in humans. FASEB J..

[4] Blusztajn J.K., Mellott T.J. (2012). Choline nutrition programs brain development via DNA and histone methylation. Central Nerv. Syst. Agents Med. Chem..

[5] Zeisel S.H. (2011). What Choline Metabolism Can Tell Us about the Underlying Mechanisms of Fetal Alcohol Spectrum Disorders. Mol. Neurobiol..

[6] Ylilauri M.P.T., Voutilainen S., Lönnroos E., Virtanen H.E.K., Tuomainen T.-P., Salonen J.T., Virtanen J.K. (2019). Associations of dietary choline intake with risk of incident dementia and with cognitive performance: The Kuopio Ischaemic Heart Disease Risk Factor Study. Am. J. Clin. Nutr..

[7] Poly C., Massaro J.M., Seshadri S., Wolf P.A., Cho E., Krall E., Jacques P.F., Au R. (2011). The relation of dietary choline to cognitive performance and white-matter hyperintensity in the Framingham Offspring Cohort. Am. J. Clin. Nutr..

[8] Vizuete A.A., Robles F., Rodríguez-Rodríguez E., López-Sobaler A.M., Ortega R.M. (2009). Association between food and nutrient intakes and cognitive capacity in a group of institutionalized elderly people. Eur. J. Nutr..

[9] Zhong C., Lu Z., Che B., Qian S., Zheng X., Wang A., Bu X., Zhang J., Ju Z., Xu T. (2021). Choline Pathway Nutrients and Metabolites and Cognitive Impairment after Acute Ischemic Stroke. Stroke.

[10] Miao M., Du J., Che B., Guo Y., Zhang J., Ju Z., Xu T., Zhong X., Zhang Y., Zhong C. (2021). Circulating choline pathway nutrients and depression after ischemic stroke. Eur. J. Neurol..

[11] Jacobson S.W., Carter R.C., Molteno C.D., Stanton M.E., Herbert J.S., Lindinger N.M., Lewis C.E., Dodge N.C., Hoyme H.E., Zeisel S.H. (2018). Efficacy of Maternal Choline Supplementation During Pregnancy in Mitigating Adverse Effects of Prenatal Alcohol Exposure on Growth and Cognitive Function: A Randomized, Double-Blind, Placebo-Controlled Clinical Trial. Alcohol. Clin. Exp. Res..

[12] Warton F.L., Molteno C.D., Warton C.M.R., Wintermark P., Lindinger N.M., Dodge N.C., Zöllei L., Kouwe A.J., Carter R.C., Jacobson J.L. (2021). Maternal choline supplementation mitigates alcohol exposure effects on neonatal brain volumes. Alcohol. Clin. Exp. Res..

[13] King J.H., Kwan S.T., Bae S., Klatt K.C., Yan J., Malysheva O.V., Jiang X., Roberson M.S., Caudill M.A. (2019). Maternal choline supplementation alters vitamin B-12 status in human and murine pregnancy. J. Nutr. Biochem..

[14] Wang Z., Klipfell E., Bennett B.J., Koeth R., Levison B.S., DuGar B., Feldstein A.E., Britt E.B., Fu X., Chung Y.-M. (2011). Gut Flora Metabolism of Phosphatidylcholine Promotes Cardiovascular Disease. Nature.

[15] He S., Jiang H., Zhuo C., Jiang W. (2021). Trimethylamine/Trimethylamine-*N*-Oxide as a Key between Diet and Cardiovascular Diseases. Cardiovasc. Toxicol..

[16] Chawla R.K., Wolf D.C., Kutner M.H., Bonkovsky H.L. (1989). Choline may be an essential nutrient in malnourished patients with cirrhosis. Gastroenterology.

[17] Sheard N.F., Tayek J.A., Bistrian B.R., Blackburn G.L., Zeisel S.H. (1986). Plasma choline concentration in humans fed parenterally. Am. J. Clin. Nutr..

[18] Zeisel S.H., da Costa K.-A. (1991). Choline, an essential nutrient for humans. FASEB J. Off. Publ. Fed. Am. Soc. Exp. Biol..

[19] Goh Y.Q., Cheam G., Wang Y. (2021). Understanding Choline Bioavailability and Utilization: First Step Toward Personalizing Choline Nutrition. J. Agric. Food Chem..

[20] Hwang J.-S., Shin Y.-J. (2021). Role of Choline in Ocular Diseases. Int. J. Mol. Sci..

[21] Wortmann S.B., Mayr J.A. (2018). Choline-related-inherited metabolic diseases—A mini review. J. Inherit. Metab. Dis..

[22] Zeisel S.H., da Costa K.-A. (2009). Choline: An essential nutrient for public health. Nutr. Rev..

[23] Derbyshire E., Obeid R., Schön C. (2021). Habitual Choline Intakes across the Childbearing Years: A Review. Nutrients.

[24] Blusztajn J.K., Slack B.E., Mellott T.J. (2017). Neuroprotective Actions of Dietary Choline. Nutrients.

[25] Zeisel S.H., Klatt K.C., Caudill M.A. (2018). Choline. Adv. Nutr. Int. Rev. J..

[26] Probst Y., Sulistyoningrum D.C., Netting M.J., Gould J.F., Wood S., Makrides M., Best K.P., Green T.J. (2022). Estimated Choline Intakes and Dietary Sources of Choline in Pregnant Australian Women. Nutrients.

[27] Zhu C., Sawrey-Kubicek L., Bardagjy A.S., Houts H., Tang X., Sacchi R., Randolph J.M., Steinberg F.M., Zivkovic A.M. (2020). Whole egg consumption increases plasma choline and betaine without affecting TMAO levels or gut microbiome in overweight postmenopausal women. Nutr. Res..

[28] Wallace T.C., Blusztajn J.K., Caudill M.A., Klatt K.C., Natker E., Zeisel S.H., Zelman K.M. (2018). Choline. Nutr. Today.

[29] Perrin M.T., Pawlak R., Allen L.H., Hampel D. (2019). Total Water-Soluble Choline Concentration Does Not Differ in Milk from Vegan, Vegetarian, and Nonvegetarian Lactating Women. J. Nutr..

[30] Molloy A.M., Mills J., Cox C., Daly S.F., Conley M., Brody L.C., Kirke P.N., Scott J.M., Ueland P.M. (2005). Choline and homocysteine interrelations in umbilical cord and maternal plasma at delivery. Am. J. Clin. Nutr..

[31] Ilcol Y.O., Ozbek R., Hamurtekin E., Ulus I.H. (2005). Choline status in newborns, infants, children, breast-feeding women, breast-fed infants and human breast milk. J. Nutr. Biochem..

[32] Efsa N.D., Panel (EFSA Panel on Dietetic Products, Nutrition and Allergies) (2016). Dietary Reference Values for choline. EFSA J..

[33] Cochrane K.M., A Williams B., Elango R., I Barr S., Karakochuk C.D. (2022). Pregnancy-induced alterations of 1-carbon metabolism and significance for maternal nutrition requirements. Nutr. Rev..

[34] Jadavji N., Deng L., Malysheva O., Caudill M., Rozen R. (2015). MTHFR deficiency or reduced intake of folate or choline in pregnant mice results in impaired short-term memory and increased apoptosis in the hippocampus of wild-type offspring. Neuroscience.

[35] Ross R.G., Hunter S.K., McCarthy L., Beuler J., Hutchison A.K., Wagner B., Leonard S., Stevens K.E., Freedman R. (2013). Perinatal Choline Effects on Neonatal Pathophysiology Related to Later Schizophrenia Risk. Am. J. Psychiatry.

[36] Wang Y., Surzenko N., Friday W.B., Zeisel S.H. (2015). Maternal dietary intake of choline in mice regulates development of the cerebral cortex in the offspring. FASEB J..

[37] Derbyshire E., Obeid R. (2020). Choline, Neurological Development and Brain Function: A Systematic Review Focusing on the First 1000 Days. Nutrients.

[38] Caudill M.A., Strupp B.J., Muscalu L., Nevins J.E.H., Canfield R.L. (2018). Maternal choline supplementation during the third trimester of pregnancy improves infant information processing speed: A randomized, double-blind, controlled feeding study. FASEB J..

[39] Obeid R., Derbyshire E., Schön C. (2022). Association between maternal choline, foetal brain development and child neurocognition; systematic review and meta-analysis of human studies. Adv. Nutr. Int. Rev. J..

[40] Irvine N., England-Mason G., Field C.J., Dewey D., Aghajafari F. (2022). Prenatal Folate and Choline Levels and Brain and Cognitive Development in Children: A Critical Narrative Review. Nutrients.

[41] Wallace T.C. (2018). A Comprehensive Review of Eggs, Choline, and Lutein on Cognition across the Life-span. J. Am. Coll. Nutr..

[42] Adams J.B., Kirby J.K., Sorensen J.C., Pollard E.L., Audhya T. (2022). Evidence based recommendations for an optimal prenatal supplement for women in the US: Vitamins and related nutrients. Matern. Health Neonatol. Perinatol..

[43] Horita D.A., Hwang S., Stegall J.M., Friday W.B., Kirchner D.R., Zeisel S.H. (2021). Two methods for assessment of choline status in a randomized crossover study with varying dietary choline intake in people: Isotope dilution MS of plasma and in vivo single-voxel magnetic resonance spectroscopy of liver. Am. J. Clin. Nutr..

[44] NHANES Questionnaires, Datasets, and Related Documentation. https://wwwn.cdc.gov/nchs/nhanes/default.aspx.

[45] Fawzi W. (2004). Calibration of a semi-quantitative food frequency questionnaire in early pregnancy. Ann. Epidemiol..

[46] Villamor E., Rifas-Shiman S.L., Gillman M.W., Oken E. (2012). Maternal Intake of Methyl-Donor Nutrients and Child Cognition at 3 Years of Age. Paediatr. Périnat. Epidemiol..

[47] Zeisel S.H., Mar M.-H., Howe J.C., Holden J.M. (2003). Concentrations of Choline-Containing Compounds and Betaine in Common Foods. J. Nutr..

[48] (1998). Institute of Medicine (US) Standing Committee on the Scientific Evaluation of Dietary Reference Intakes and Its Panel on, Choline.

[49] Wiedeman A.M., Barr S.I., Green T.J., Xu Z., Innis S.M., Kitts D.D. (2018). Dietary Choline Intake: Current State of Knowledge Across the Life Cycle. Nutrients.

[50] Vennemann F.B.C., Ioannidou S., Valsta L.M., Dumas C., Ocké M.C., Mensink G.B.M., Lindtner O., Virtanen S.M., Tlustos C., D’Addezio L. (2015). Dietary intake and food sources of choline in European populations. Br. J. Nutr..

[51] Bahnfleth C., Canfield R., Nevins J., Caudill M., Strupp B. (2019). Prenatal Choline Supplementation Improves Child Color-location Memory Task Performance at 7 Y of Age (FS05-01-19). Curr. Dev. Nutr..

[52] Wallace T.C., Fulgoni V.L. (2017). Usual Choline Intakes Are Associated with Egg and Protein Food Consumption in the United States. Nutrients.

[53] Taesuwan S., McDougall M.Q., Malysheva O.V., Bender E., Nevins J.E.H., Devapatla S., Vidavalur R., Caudill M.A., Klatt K.C. (2021). Choline metabolome response to prenatal choline supplementation across pregnancy: A randomized controlled trial. FASEB J..

[54] Imbard A., Benoist J.-F., Blom H.J. (2013). Neural Tube Defects, Folic Acid and Methylation. Int. J. Environ. Res. Public Health.

[55] Hammoud R., Pannia E., Kubant R., Wasek B., Bottiglieri T., Malysheva O.V., Caudill M.A., Anderson G.H. (2021). Choline and Folic Acid in Diets Consumed during Pregnancy Interact to Program Food Intake and Metabolic Regulation of Male Wistar Rat Offspring. J. Nutr..

[56] Radziejewska A., Chmurzynska A. (2018). Folate and choline absorption and uptake: Their role in fetal development. Biochimie.

[57] Wallace T.C., Blusztajn J.K., Caudill M.A., Klatt K.C., Zeisel S.H. (2019). Choline: The Neurocognitive Essential Nutrient of Interest to Obstetricians and Gynecologists. J. Diet. Suppl..

[58] Shim J.-S., Oh K., Kim H.C. (2014). Dietary assessment methods in epidemiologic studies. Epidemiol. Health.

[59] Willemse J.P.M.M., Meertens L.J.E., Scheepers H.C.J., Achten N.M.J., Eussen S.J., van Dongen M.C., Smits L.J.M. (2019). Calcium intake from diet and supplement use during early pregnancy: The Expect study I. Eur. J. Nutr..

[60] Hjartåker A., Andersen L.F., Lund E. (2007). Comparison of diet measures from a food-frequency questionnaire with measures from repeated 24-hour dietary recalls. The Norwegian Women and Cancer Study. Public Health Nutr..

