# Supplementary material for: Inadequate Choline Intake in Pregnant Women in Germany
Source: Nutrients. 2022 Nov 17;14(22):4862. doi: 10.3390/nu14224862 (PMC9696170; doi:10.3390/nu14224862)
Supplement: Supplementary file 1 [file nutrients-14-04862-s001.zip › nutrients-1991343-supplementary.pdf]

Supplementary Materials

Figure S1: Derivation of study sample.

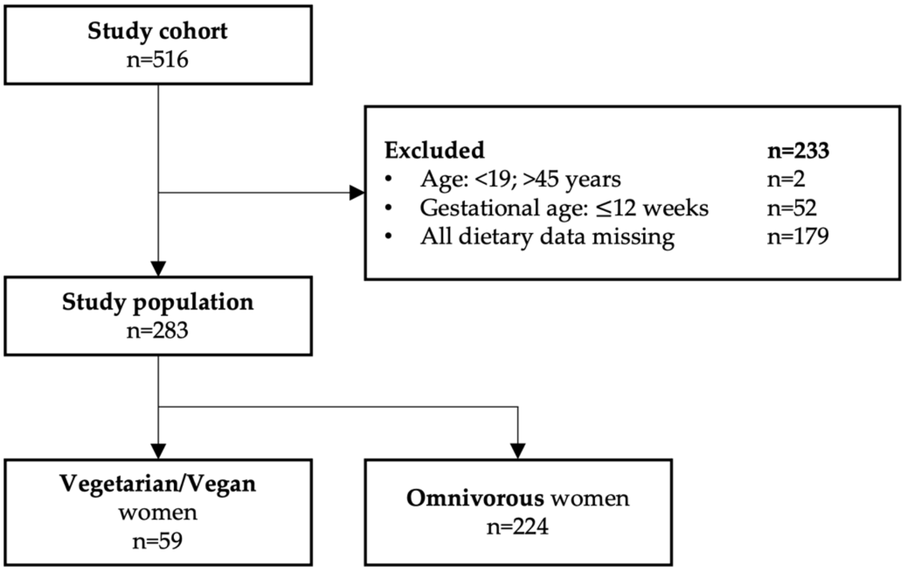

Figure S2: Original questionnaire in German language with an example of the drop-down list used for the food frequency questionnaire.

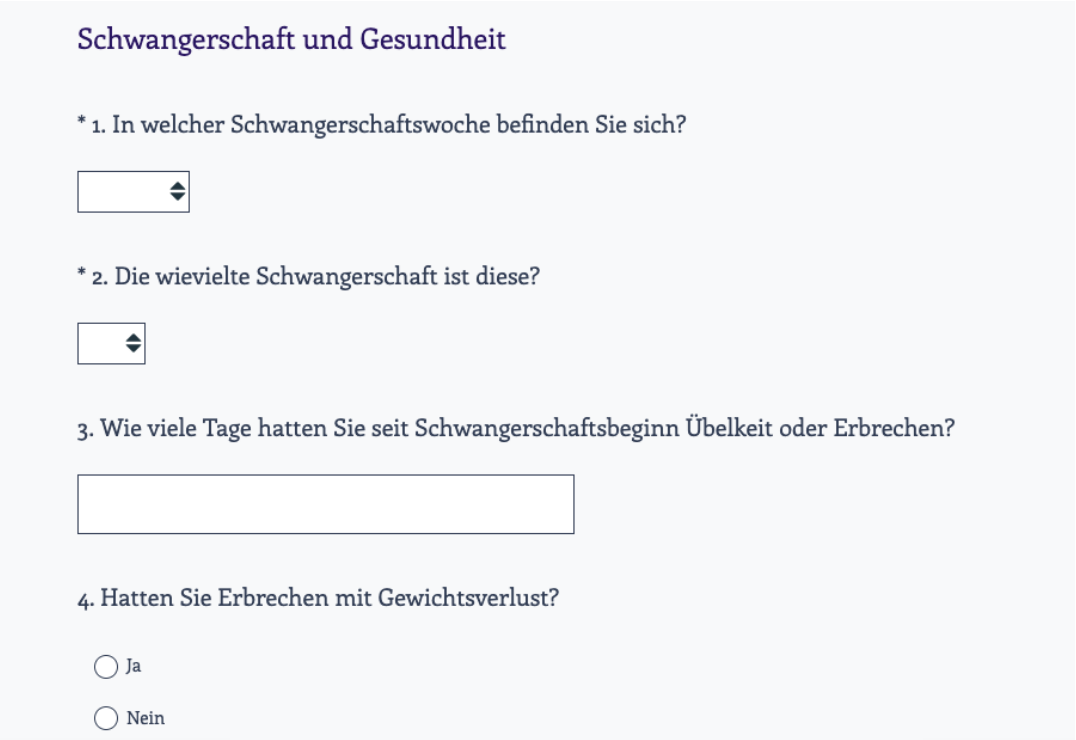
Schwangerschaft und Gesundheit

* 1. In welcher Schwangerschaftswoche befinden Sie sich?

* 2. Die wievielte Schwangerschaft ist diese?

3. Wie viele Tage hatten Sie seit Schwangerschaftsbeginn Übelkeit oder Erbrechen?

4. Hatten Sie Erbrechen mit Gewichtsverlust?

- Ja
- Nein

Ernährung und Ernährungsverhalten

* 5. Ernähren Sie sich nach einer besonderen Ernährungsweise?

- ◯ Nein
- ◯ Besondere Ernährung wegen Nahrungsmittelunverträglichkeit(en)
- ◯ Vegetarisch
- ◯ Vegan
- ◯ Sonstiges (bitte angeben)

6. Welche Nahrungsmittelunverträglichkeit(en) haben Sie?

7. Haben Sie Ihre Ernährung aufgrund der Schwangerschaft umgestellt?

- ◯ Nein
- ◯ Ja, weil

8. Welche der folgenden Vitamine und Mineralstoffe nehmen Sie seit der Feststellung der Schwangerschaft bzw. seit Beginn des Kinderwunsches in Form von Tabletten, Pulvern oder Säften zu sich?

- ☐ Keine
- ☐ Vitamin A
- ☐ Vitamin B1
- ☐ Vitamin B2
- ☐ Vitamin B6
- ☐ Vitamin B12
- ☐ Vitamin D
- ☐ Folsäure
- ☐ Eisen
- ☐ Jod
- ☐ Magnesium
- ☐ Calcium
- ☐ Sonstiges (bitte angeben)

* 9. Nehmen bzw. nahmen Sie cholinhaltige Nahrungsergänzungsmittel zu sich?

- ◯ Nein
- ◯ Ja (Bitte geben Sie den Namen des Nahrungsergänzungsmittels ein)

10. Seit wann nehmen bzw. nahmen Sie cholinhaltige Nahrungsergänzungsmittel zu sich? *(Mehrfachnennungen möglich!)*

- ☐ Vor Beginn der Schwangerschaft
- ☐ Im ersten Trimester (1. - 12. SSW)
- ☐ Im zweiten Trimester (13. - 28. SSW)
- ☐ Im dritten Trimester (29. - 40. SSW)

11. Wie oft nehmen Sie cholinhaltige Nahrungsergänzungsmittel zu sich?

- ◯ Weniger als einmal die Woche
- ◯ 1x/ Woche
- ◯ 2x/ Woche
- ◯ 3x/ Woche
- ◯ 4x/ Woche
- ◯ 5x/ Woche
- ◯ 6x/ Woche
- ◯ Täglich
- ◯ 2-3x/ täglich
- ◯ Sonstiges (bitte angeben)

12. Wie viel mg Cholin enthalten eine Dosis Ihres Nahrungsergänzungsmittels?

* 13. Wurde Ihnen die Einnahme von cholinhaltigen Nahrungsergänzungsmitteln empfohlen?

- ◯ Ja
- ◯ Nein

14. Von wem wurde ihnen die Einnahme von cholinhaltigen Nahrungsergänzungsmittel empfohlen?

- ◯ Ärztin/ Arzt
- ◯ Hebamme
- ◯ Heilpraktiker*in
- ◯ Ernährungsfachkraft
- ◯ Bekannte/ Verwandte
- ◯ Sonstige (bitte angeben)

Verzehrshäufigkeiten von Lebensmitteln

Wie oft haben Sie die folgenden Lebensmittel im Verlauf der letzten Woche zu sich genommen?

Bitte richten Sie sich nach den angegebenen Portionsgrößen beim Einschätzen des wöchentlichen Verzehrs.
Eine Portion entspricht dabei den angezeigten Mengen. Sollten Portionsgrößen nicht in dem Bild zu sehen sein, ist eine weitere Beschreibung unterhalb des Bildes zu finden.
Wenn Sie sich nicht ganz sicher sind, schätzen Sie bitte.
Viel Spaß beim weiteren Ausfüllen des Fragebogens.

* 15. Getränke

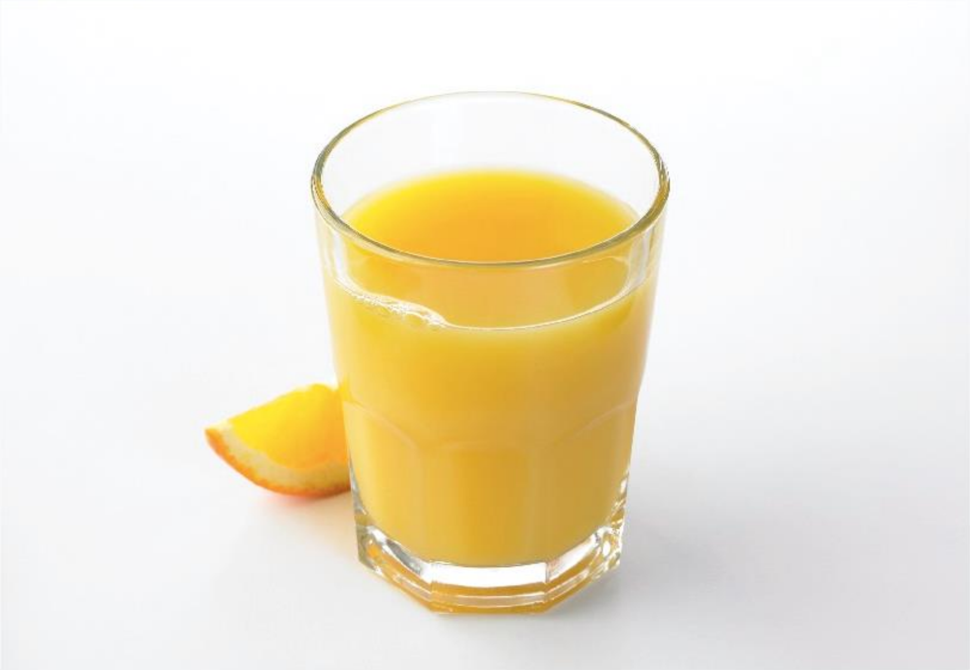

Eine Portion entspricht einem Glas von 200ml.

	Glas/ Gläser
Zuckerhaltige Erfrischungsgetränke z.B. Cola, Malzbier	
Fruchtsaft aus z.B. Orange, Apfel	
Gemüsesaft aus z.B. Karotte, Tomate	
Früchte-/ Kräutertee	

* 16. Getreide-/ Vollkornprodukte

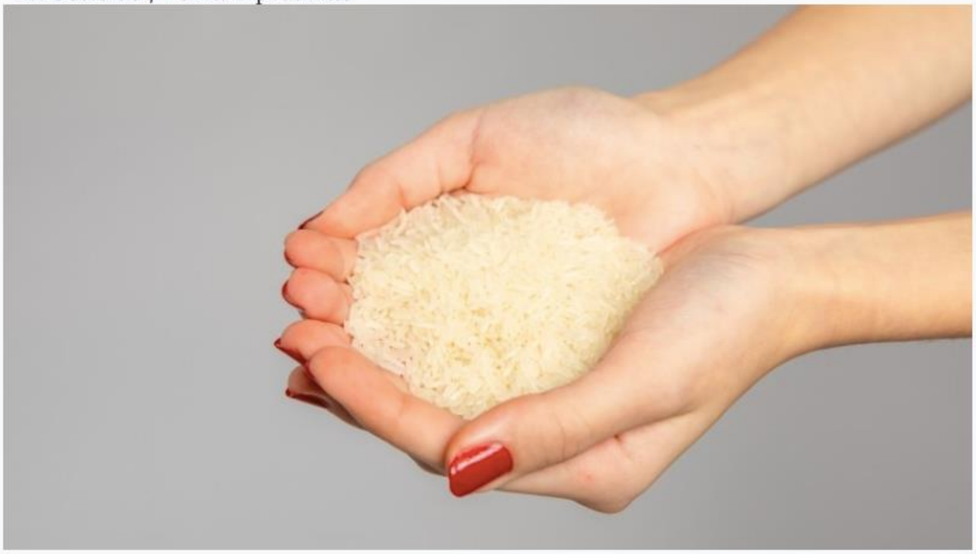

Eine Scheibe Brot bedeckt die gesamte Handfläche mit ausgestreckten Fingern.

	P[illegible]
Cornflakes	
Müsli	
Scheibe Vollkornbrot/ Vollkornbrötchen	
Scheibe Graubrot/ Mischbrot	
Scheibe Weißbrot/ Brötchen	
Reis	
Getreide z.B. Couscous oder Bulgur	
Nudeln	
Kartoffeln	

8
9
10
11
12
13
14
15
16
17
18
19
20
21
22
23
24

* 17. Brotbelag

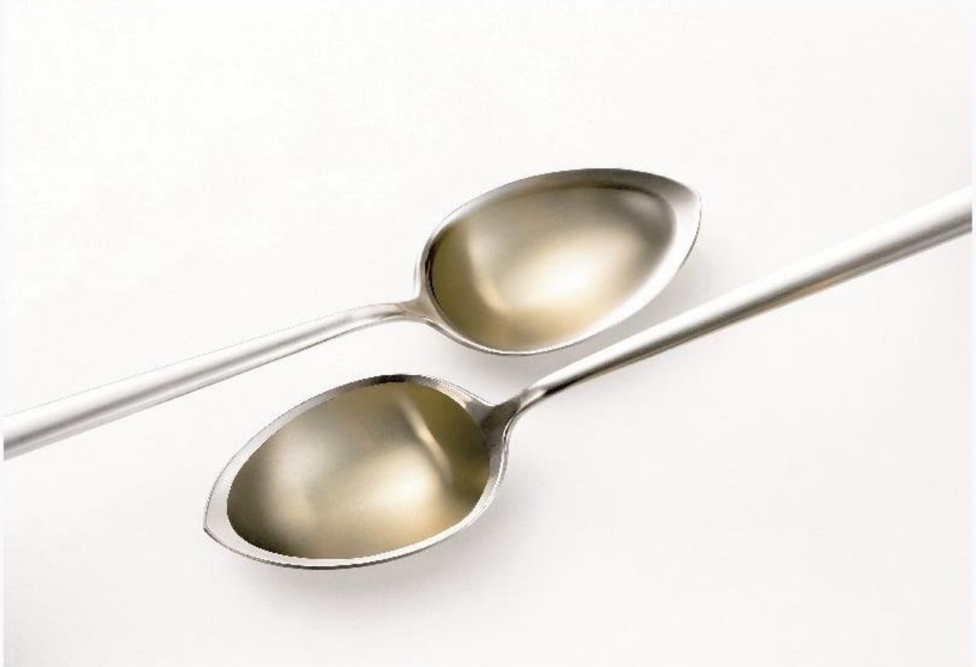

Eine Portion entspricht einem Esslöffel bzw. einer Scheibe.

	Portion(en)
Butter oder Margarine	
Frischkäse	
Käse	
vegetarische/ vegane Streichcreme	
Salami	
Schinken	
Fleischwurst	
Honig oder Marmelade	
Nusscreme z.B. Erdnusscreme, Nuss-Nougatcreme	

* 18. Milchprodukte und Eier

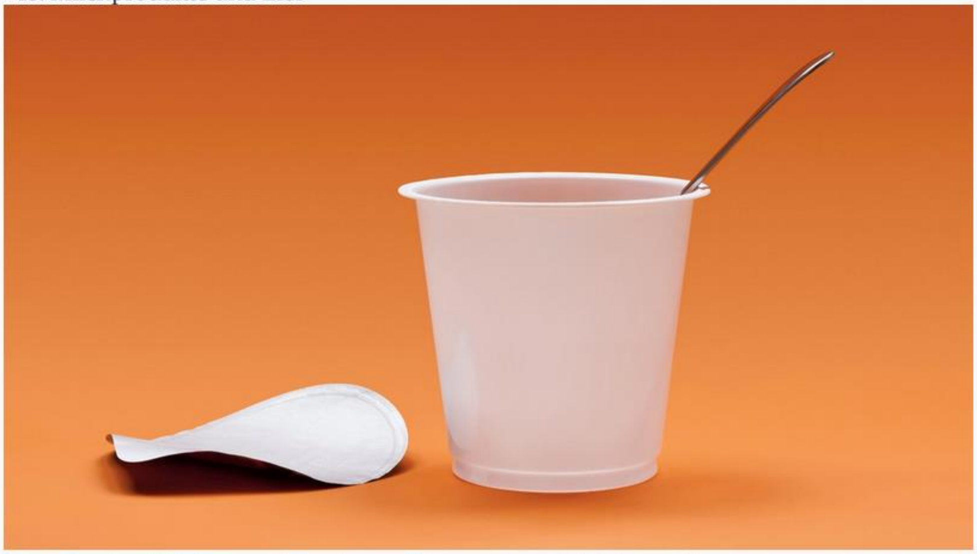

Eine Portion Milch entspricht einem Glas von 200ml (s. Frage 16).

	Portion(en)
Ein Glas Milch	
Ein Becher (125g) Quark/ Joghurt/ Dickmilch	
Ei z.B. Spiegelei, Rührei	

* 19. Fleisch/ -waren

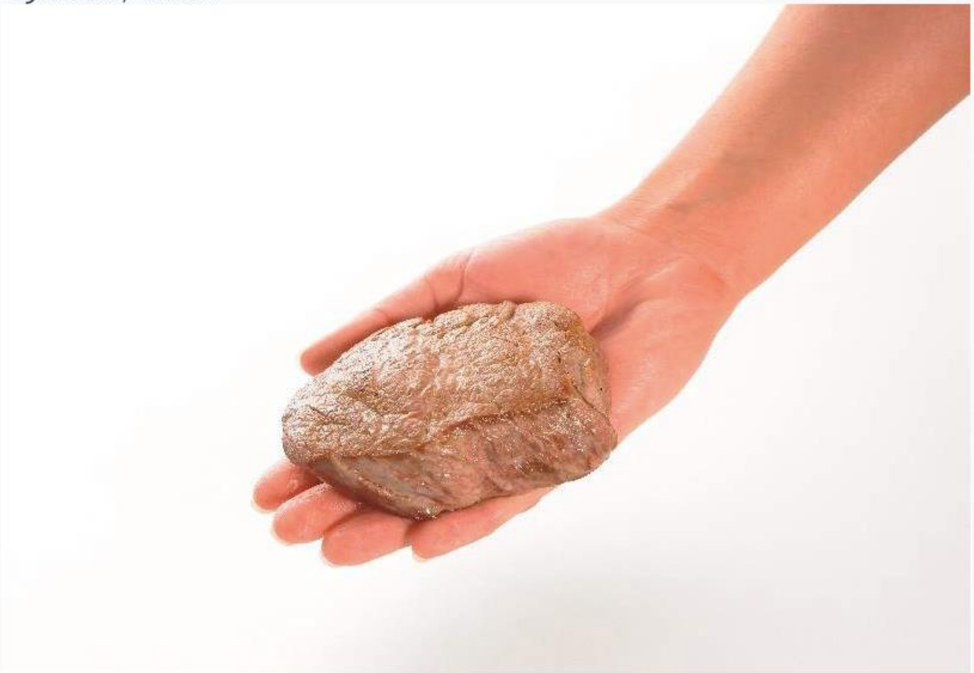

Eine Portion Fleisch entspricht der Größe des Handtellers.

	Portion(en)
Weißes Fleisch z.B. Hähnchen	
Rotes Fleisch z.B. Schweinefleisch, Rindfleisch	
Innereien z.B. Leber, Niere	

* 20. Fisch und Meeresfrüchte

Eine Portion entspricht der Größe des Handtellers (s. Frage 20).

	Portion(en)
Fettreicher Fisch z.B. Lachs, Thunfisch	
Fettarmer Fisch z.B. Scholle, Forelle	
Krustentiere z.B. Krabben, Krebs	
Muscheln	

* 21. Obst

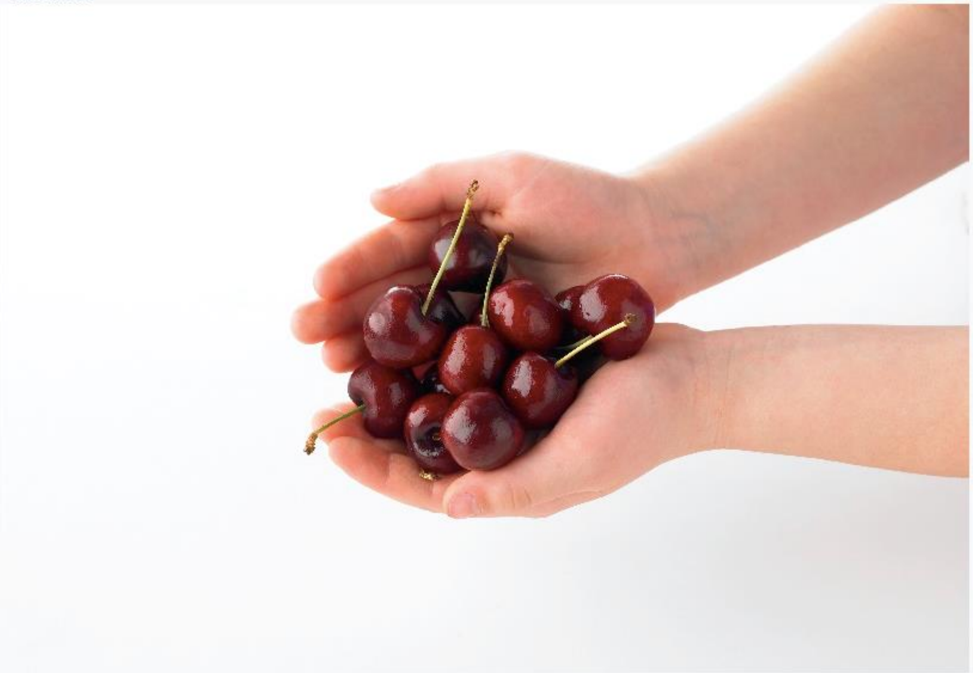

Eine Portion entspricht zwei Hände zu einer Schale gehalten für zerkleinertes oder kleinstückiges Obst/ Gemüse oder eine Portion entspricht einem Stück Obst/ Gemüse (s. Frage 23).

	Portion(en)
Kernobst z.B. Apfel, Birne	
Steinobst z.B. Kirsche, Marille	
Beeren z.B. Erdbeeren, Himbeere	
Südfrüchte z.B. Banane, Kiwi	

* 22. Gemüse

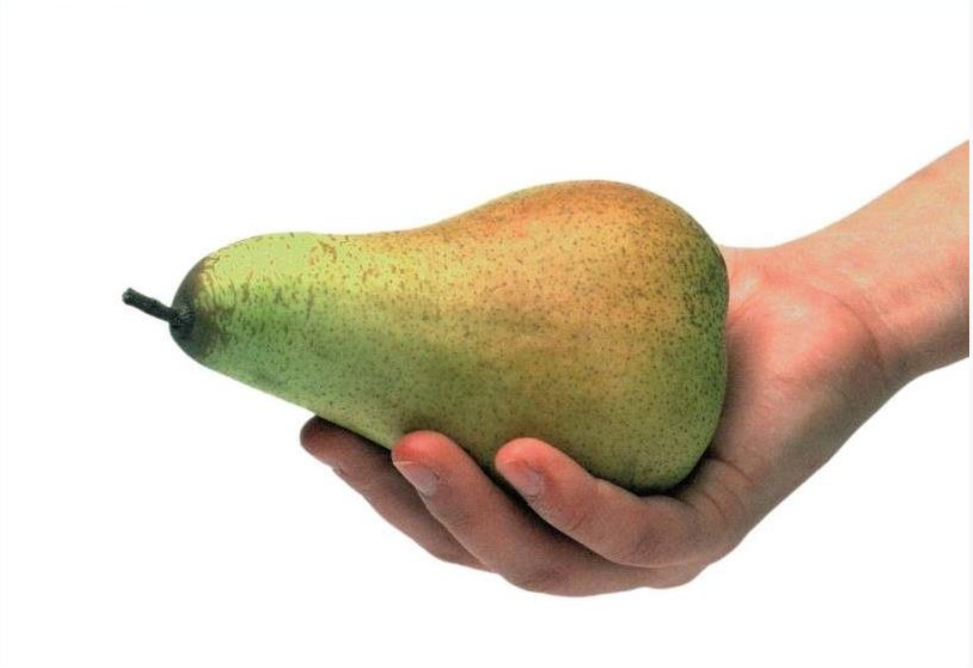

Eine Portion entspricht einem Stück Obst/ Gemüse oder zwei Hände zur Schale gehalten für zerkleinertes oder kleinstückiges Obst/ Gemüse (s. Frage 22).

	Portion(en)
Weißes Kohlgemüse z.B. Weißkohl, Chinakohl	
Grünes Kohlgemüse z.B. Brokkoli, Rosenkohl	
Diverse Gemüse z.B. Tomate, Gurke, Paprika	
Pilze	

* 23. Hülsenfrüchte

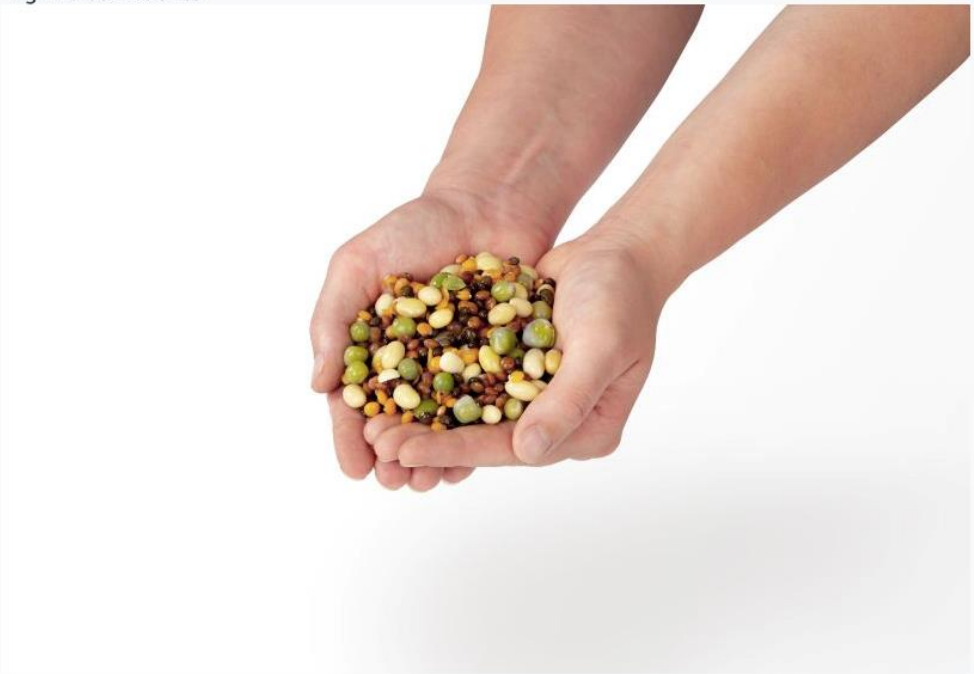

Eine Portion Hülsenfrüchte passt in zwei Hände.

	Portion(en)
Brechbohnen	
Tofu	
Erbsen	
Linsen	
Bohnensamen	

* 24. Nüsse und Samen

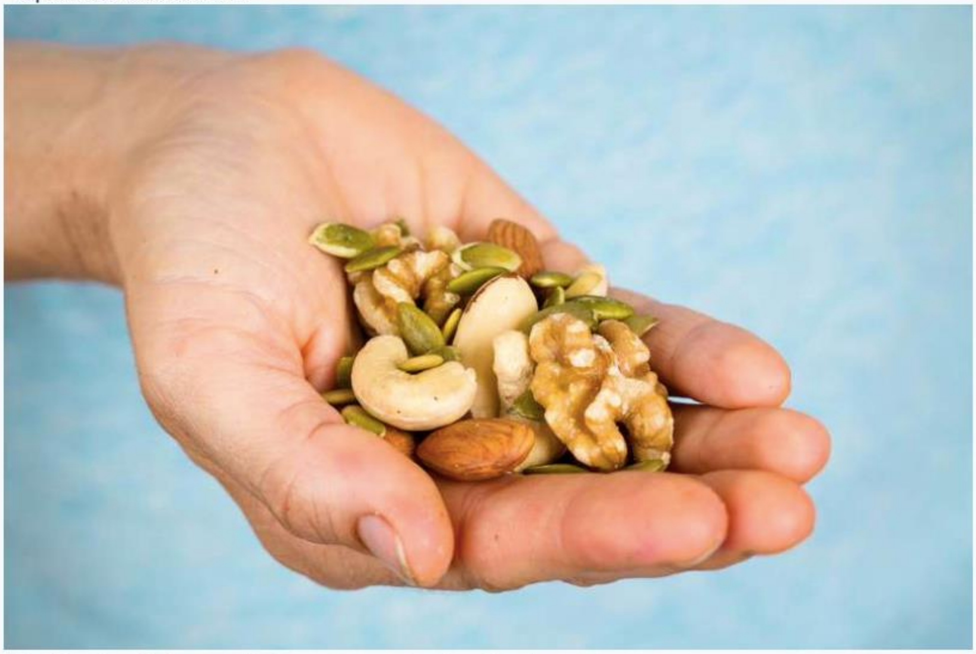

Eine Portion Nüsse und Samen passt in eine Hand.

	Portion(en)
Sesam	
Walnuss	
Paranuss	
Macadamianuss	
Haselnuss	
Erdnuss	
Sonnenblumenkerne	
Pinienkerne	
Cashewnuss	

* 25. Sonstiges

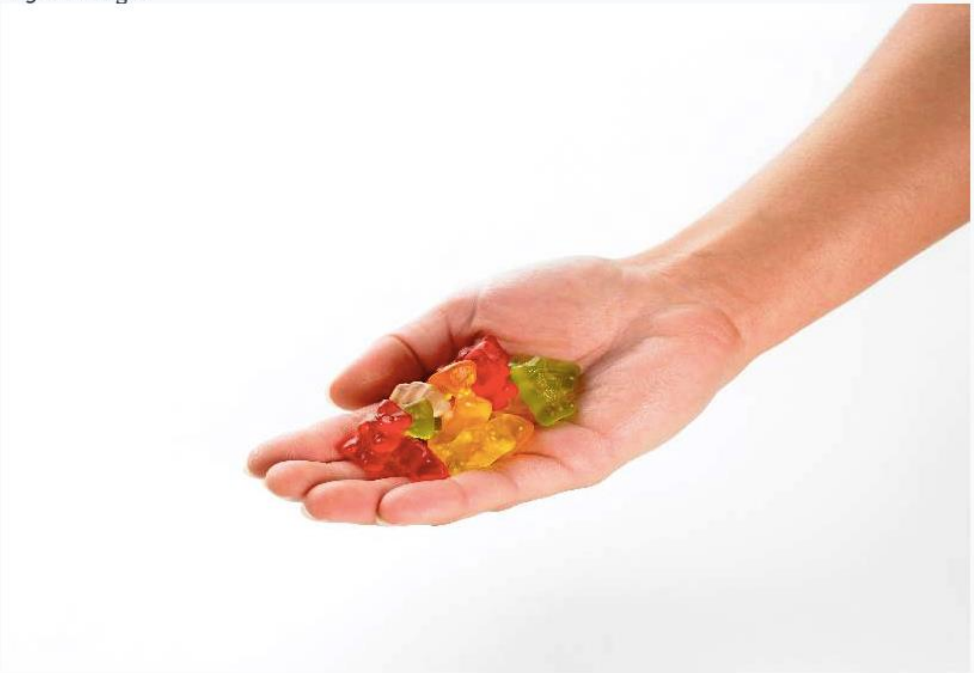

Eine Portion Süßigkeiten passt in eine Hand.

	Portion(en)
Eine Hand Süßigkeiten z.B. Schokolade, Gummibärchen	
Ein Stück Kuchen oder Torte	
Fast Food z.B. eine Pizza, ein Hamburger	

* 26. Fette

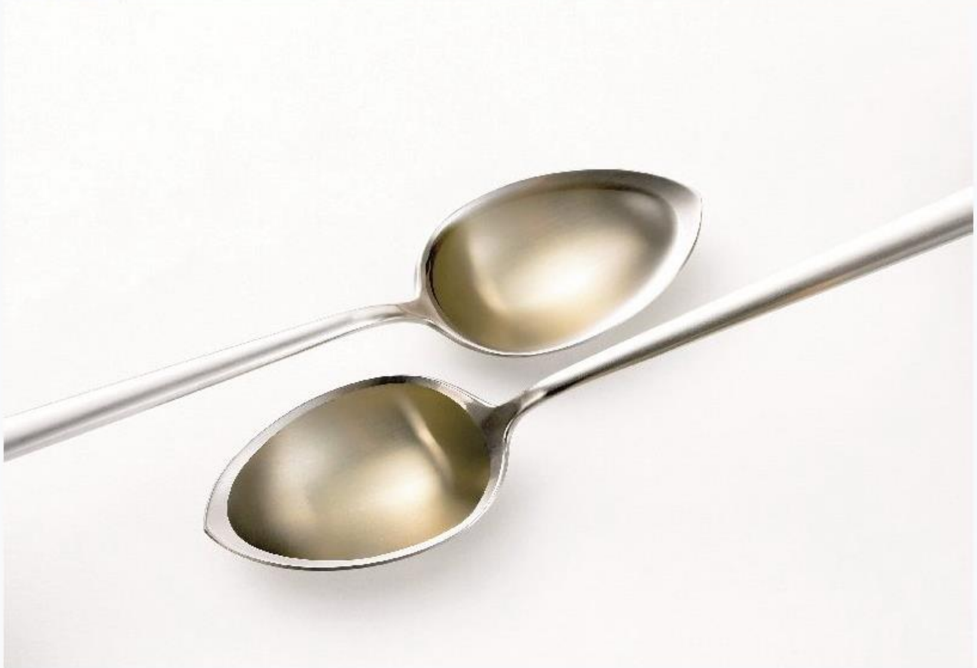

Eine Portion entspricht <u>zwei</u> Esslöffeln

	Portion(en)
Butter/ Margarine	
Olivenöl	
Pflanzliches Kochfett z.B. Baskin, Palmin	
Tierisches Kochfett z.B. Schmalz	
Sonnenblumenöl	
Weizenkeimöl	
Sonstiges	

Sonstiges (bitte angeben)

Angaben zur Person

* 27. Wie alt sind Sie?

- ◯ < 19
- ◯ 19-25
- ◯ 26-30
- ◯ 31-35
- ◯ 36-40
- ◯ 41-45
- ◯ > 45

28. Wie ist Ihre familiäre Situation?

- ◯ Lebe alleine (ledig, geschieden,...)
- ◯ Lebe mit meinem/meiner Partner*in
- ◯ Lebe mit Familie (Partner*in, Kind(er),...)
- ◯ Sonstiges (bitte angeben)

29. Was ist Ihr höchster erreichter Bildungsabschluss?

- ◯ (Noch) keinen Schulabschluss
- ◯ Hauptschulabschluss
- ◯ Realschulabschluss (Mittlere Reife, Fachoberschulreife, o.Ä.)
- ◯ Sonstiges (bitte angeben)
- ◯ Abitur (allgemeine Hochschulreife), fachgebundene Hochschulreife oder Fachhochschulreife
- ◯ (Fach-) Hochschulabschluss (Bachelor, Master, Magister, Diplom, Staatsexamen, Promotion)

* 30. In welchem Bundesland wohnen Sie?

Ende!

Vielen Dank, dass Sie sich die Zeit genommen haben den Fragebogen auszufüllen.
Wir wünsche Ihnen alles Gute für die weitere Schwangerschaft.

Translated version with summary of results

Questionnaire

1. Health and Pregnancy

Table 1-4: Translated questionnaire. Distribution of whole study population is shown in the tables. Values are numbers and percentages according to categories specified in the first column.

**What gestational week are you in?**	
13-41	283 (100%)
**How many pregnancies did you have before this one?**	
1.	134 (47.35%)
2.	87 (30.74%)
3.	44 (15.55%)
4.	9 (3.18%)
5.	4 (1.41%)
6.	1 (0.35%)
7.	2 (0.71%)
8.	2 (0.71%)
**How many days have you had nausea or vomiting since the beginning of your pregnancy?**	
**Did you lose weight due to vomiting?**	
Yes	54 (19.15%)
No	228 (80.85%)

2. Dietary habits and use of dietary supplements

**Do you follow one of the following diets?**	
No	207 (73.14%)
Certain diet due to intolerances	7 (2.47%)
Vegetarian	43 (15.19%)
Vegan	13 (4.59%)
Others (please specify)	13 (4.59%)
**What kind of intolerances?**	
Open question	
**Did you change your dietary habits due to pregnancy?**	
No	227 (80.21%)
Yes	56 (19.79%)
**Which of the following vitamins and minerals have you been taking in the form of tablets, powders or juices since you became pregnant or started trying to have children?**	
None	9 (3.20%)
Vitamin A	49 (17.44%)
Vitamin B1	81 (28.83%)
Vitamin B2	83 (29.54 %)
Vitamin B6	94 (33.45%)
Vitamin B12	139 (49.47%)
Vitamin D	146 (51.96%)
Folic Acid	256 (91.10%)
Iron	111 (39.50%)
Iodine	142 (50.53%)
Magnesium	139 (49.47%)
Calcium	62 (22.06%)

Others (please specify)	58 (20.64%)
**Do you take or have taken choline-containing supplements?**	
No	267 (94.35%)
Yes	16 (5.65%)
**If you take choline-containing supplements, since when? Multiple answers possible**	
Before pregnancy	5 (33.33%)
First trimester	10 (66.67%)
Second trimester	9 (60.00%)
Third trimester	6 (40.00%)
**How often do you take choline-containing supplements?**	
Less than once a week	2 (12.50%)
1 per week	0
2 per week	0
3 per week	1 (6.25%)
4 per week	2 (12.50%)
5 per week	0
6 per week	0
Daily	10 (62.50%)
2-3 times per day	0
Others (please specify)	1 (6.25%)
**How much choline contains one dose?**	
Open question	
**Have you been advised to take choline containing supplements?**	
No	280 (98.94%)
Yes	3 (1.06%)
**If yes, who advised it?**	
Doctor	1 (33.33%)
Midwife	0
Alternative practitioner	1 (33.33%)
Nutritional specialist	1 (33.33%)
Friends or Relatives	0
Others (please specify)	0

3. Food Frequency Questionnaire

How often did you consume the following food items/ groups within the last week?

Please refer to shown hand portion sizes when you estimate your weekly consumption. If there is no respective portion presented in the picture, please refer to the description along the picture. In case of uncertainty, please estimate appropriately.

All the best for the rest of the questionnaire.

**Drinks**	
Sugary Soft Drinks	Choose between 0-30 portions
Fruit juice	
Vegetable juice	
Tea	
**Cereals or wholemeal products**	
Cornflakes	
Muesli	
Whole grain bread	
Rye bread	

White bread	
Rice	
Grains like couscous	
Pasta	
Potatoes	
**Bread topping**	
Butter or margarine	
Cream cheese	
Cheese	
Vegetarian/ vegan alternatives	
Salami	
Ham	
Pork sausage	
Honey or jam	
Nut cream like peanut butter	
**Milk products and eggs**	
Milk (200 ml)	
Yoghurt (125 g)	
Egg	
Meat and meat products	
White meat like poultry	
Red meat	
Offal like liver, kidneys …	
**Fish and Sea Food**	
Fatty fish	
Lean fish	
Crustacea	
Mussels	
**Fruits**	
Pome fruit like apples and pears	
Stone fruit like cherry	
Berries	
Southern fruits	
**Vegetable**	
White kale	
Green kale	
Diverse vegetables	
Mushrooms	
**Legumes**	
Green beans	
Tofu	
Peas	
Lentils	
Beanseeds	
**Nuts and seeds**	
Sesame	
Walnuts	
Brazilnut	
Macadamia	

Hazelnut	
Peanut	
Sunflower seeds	
Pines nuts	
Cashew	
Others	
**Sweets**	
Cake	
Fast Food	
**Fats**	
Butter margarine	
Olive oil	
Plant based cooking fat	
Animal based cooking fat	
Sunflower oil	
Wheatgerm oil	
Others	

4. Background information

**How old are you?**	
<19	0
19 – 25	17 (6.01%)
26 – 30	122 (43.11%)
31 – 35	112 (39.58%)
36 – 40	29 (10.25%)
41 – 45	3 (1.06%)
>45	0
**How is your family situation?**	
Live alone	3 (1.06%)
Live with partner	140 (49.47%)
Live with family	138 (48.76%)
Others (please specify)	2 (0.71%)
**What is your highest educational degree?**	
No degree	0
Secondary School Degree	4 (1%)
Middle School Degree	56 (20%)
High School Degree	74 (26%)
University degree	144 (51%)
Others (please specify)	5 (2%)
**In which district do you live in Germany?**	
